# The Sunflower

**Published:** 1881-06

**Authors:** 


The Sunflower.—The value of this plant is not widely known. Its seeds make the most excellent food for poultry, increasing their supply of eggs as well as fattening the chickens rapidly. It is a very healthful plant, as it absorbs the bad air of barn-yards and drains swamps.—*Floral Cabinet.*

